# The Influence of Print Orientation and Discontinuous Carbon Fiber Content on the Tensile Properties of Selective Laser-Sintered Polyamide 12

**DOI:** 10.3390/polym17152028

**Published:** 2025-07-25

**Authors:** Jonathan J. Slager, Joshua T. Green, Samuel D. Levine, Roger V. Gonzalez

**Affiliations:** 1 Department of Mechanical and Nuclear Engineering, School of Engineering, Computing and Weapons, United States Naval Academy, Annapolis, MD 21402, USA; 2 Department of Engineering Education and Leadership, College of Engineering, The University of Texas at El Paso, El Paso, TX 79968, USA; 3 Undersea Warfare Academic Group, Naval Postgraduate School, Monterey, CA 93943, USA

**Keywords:** additive manufacturing, powder bed fusion, selective laser sintering, discontinuous fiber, polymer matrix composite, anisotropy, tensile strength, functionally graded materials

## Abstract

Discontinuous fibers are commonly added to matrix materials in additive manufacturing to enhance properties, but such benefits may be constrained by print and fiber orientation. The additive processes of forming rasters and layers in powder bed fusion inherently cause anisotropy in printed parts. Many print parameters, such as laser, temperature, and hatch pattern, influence the anisotropy of tensile properties. This study characterizes fiber orientation attributed to recoating non-encapsulated fibers and the resulting anisotropic tensile properties. Tensile and fracture properties of polyamide 12 reinforced with 0%, 2.5%, 5%, and 10% discontinuous carbon fibers by volume were characterized in two primary print/tensile loading orientations: tensile loading parallel to the recoater (“horizontal specimens”) and tensile load along the build axis (“vertical specimens”). Density and fractographic analysis indicate a homogeneous mixture with low porosity and primary fiber orientation along the recoating direction for both print orientations. Neat specimens (zero fiber) loaded in either direction have similar tensile properties. However, fiber-reinforced vertical specimens have significantly reduced consistency and tensile strength as fiber content increased, while the opposite is true for horizontal specimens. These datasets and results provide a mechanism to tune material properties and improve the functionality of selectively laser-sintered fiber-reinforced parts through print orientation selection. These datasets could be used to customize functionally graded parts with multi-material selective laser-sintering manufacturing.

## 1. Introduction

Additive manufacturing (AM) has become a critical tool within many industries [1]. Polymer-based AM is often one of the most cost-effective AM methods. This cost savings is especially true when compared to metal-based AM; however, polymer parts frequently cannot meet the thermal or strength requirements of some applications. Selective Laser Sintering (SLS) is one of the most widely used polymer-based AM methods to create end-use parts [2,3]. Polyamide 12 (PA12) was selected as the matrix material in this research because of its versatility in SLS [4,5,6,7,8]. PA12 is often used in SLS due to its powder re-use ratio, cost, large sintering window, and low water absorption [6,9]. This research investigates the effects of mechanically blending carbon fiber (CF) into polyamide-12 (PA12) powder to increase the strength and stiffness of the end-use part [10]. Though SLS can be an efficient method to manufacture carbon fiber-reinforced polymer composites (CFRP), anisotropy has also long plagued AM and SLS [11,12,13] and adding fibers to create a composite material can increase anisotropy [10,14,15]. This study quantifies the anisotropic effects of adding fibers into SLS manufacturing procedures.

Mechanically blending powders and fillers in Powder Bed Fusion (PBF) has had initial success involving Polyamide-11 (PA11) with glass or silica particulates, 316 stainless steel with copper alloy, tungsten carbide with Cobalt, and Polyetheretherketone (PEEK) with Hydroxyapatite (HA) [16,17,18,19,20]. These studies demonstrate the feasibility of tuning material properties using PBF with different ratios of mechanically blended fillers or fibers. Successful SLS with blended polymer-fiber materials largely rests on two broad print processes. First, the thermal process produces particle-particle coalescence combined with particle-fiber adherence. Second, the additive nature of AM includes layer application, print orientation, and hatch pattern.

Thermally suitable polymers for SLS are limited by the distinctive and somewhat rare material properties required to coalesce solid particles via a directed energy source [4]. When sintering material blends, all materials in the blend are subject to the same energy sources and thermal conditions and, at a minimum, must be tolerant of the sintering process. Polymer blends with fibers can also be susceptible to conglomerating and clumping during powder spreading. Avoiding particle conglomeration while ensuring adhesion between blended particles, laser hatch lines, and layers during sintering is essential to final part integrity.

Within AM, PBF has unique challenges and requirements when blending powders to tune material properties compared to multi-material fabrication in other AM processes, such as Material Extrusion (MEX) or Polyjet printing [21,22,23,24,25,26,27]. Material blending is conducted in a molten or liquid state in most other manufacturing methods. This inherently requires the blend to flow in a liquid state, which becomes problematic for high percentages of fibers or particulates in composite blends. To aid in powder-fiber flowability, liquid-state blending can be conducted to encapsulate the fiber within the polymer powder particles [28]. This method normally requires re-powderization, post-blend solidification, and specialized manufacturing methods to adhere or encapsulate different fillers into a single multi-material powder mixture [29]. These methods limit tuning fiber density (e.g., cannot simply mechanically blend together any ratio of polymer and fiber) and may limit some fiber orientation benefits in mechanically mixing the fiber and polymer. Tuning fiber density to customize material properties could be integrated into emerging multi-material AM methods to create functionally graded materials (FGM). This research investigated the feasibility and effects of SLS blend ratio and print orientation on mechanically blended PA12-CF mixtures.

Blended SLS composites required some commonality in extrinsic and optical properties between the materials to ensure uniform sintering [4,10]. These mutual optical properties can facilitate polymer-fiber coalescence and fusion through the inherent SLS thermal process of selectively softening the powder through energy addition. Grey PA12 powder is a semi-crystalline thermoplastic and is a widely used SLS polymer that also has thermal properties compatible with CF [4,7]. Semi-crystalline thermoplastics begin to soften (rapid decrease in modulus of elasticity) when approaching the material’s glass transition (Tg) [30]. Polymer chains possess sufficient thermal energy to mobilize at Tg when viscosity dominates. At this point, the polymer chains can begin to entangle around the fiber. However, substantial softening does not occur until the melting temperature (Tm) is reached, at which point the crystalline regions lose their structure [31] and the polymer chains fully entangle the CF. Polymer-sintering processes are complex, but primary binding mechanisms occur during cooldown. Polymers stiffen at the re-crystallization temperature (Tr) when the chains reposition and cross-link and can solidify around CF. Laser energy absorption for CF is typically over 70% with minimal reflection at a wavelength of ~455 nm [32]. High absorption and low reflectance of CF can achieve uniform heating around PA12 powder and aid coalescence of the PA12 matrix and bonding between PA12 and CF. However, CFs will not bond directly to each other, which can produce large stress concentrations where fibers are in direct contact in the final part.

**Figure 1:**
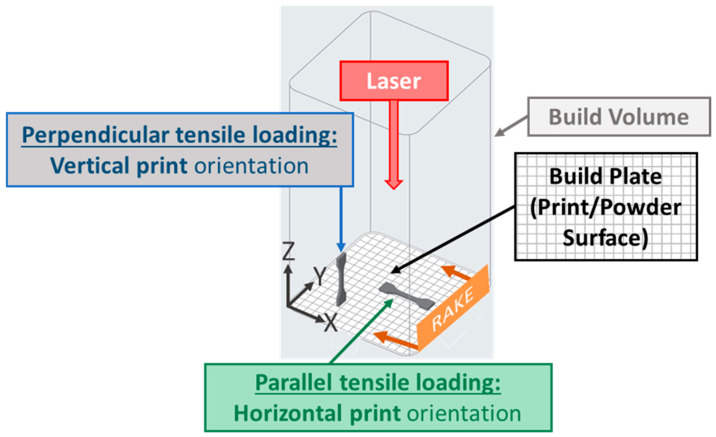
Loading and print orientation designations for Type V tensile test specimens.

The additive nature of SLS also influences final part properties. Differences in coalescence between rasters and layers inherently lead to anisotropy in printed parts [33]. Anisotropic tensile properties are influenced by laser, temperature, and hatch print parameters. However, the greatest impact on tensile properties is generally based on the following two primary loading directions (Figure 1) [34,35]. First, perpendicular tensile loading (“vertically” printed)—tensile load along the build direction transmits the load perpendicularly to the printed layers or from layer to layer resulting in interlayer loading. Vertically printed tensile specimens reduce the sintered area for each layer and, as a result, generally have reduced part warping and sledding as compared to the horizontal print orientation. Second, parallel tensile loading (“horizontally” printed)—tensile load parallel to the layers transmits the load within or along layers, resulting in intralayer loading. Horizontally printed tensile specimens decrease the number of layers from ~635 to ~32 when compared with the vertically printed specimens. This greatly decreases the print time and, thus, the time the reservoir powder remains at elevated temperatures.

The print orientation also influences the alignment between fibers and the load direction. Fibers tend to orient along the direction of the recoater which increases strength in this direction. However, the orientation of the fibers can also affect the sintering of the material because fibers may reflect and/or absorb energy differently than the polymer matrix material. This may decrease or alter polymer sintering around fibers. Multi-recoating and multi-lasing were used in this study to mitigate these effects [36].

The PBF and stock material factors discussed (blending, shape, spreading, orientation, reflectivity, absorptivity, print time, etc.) add complexity to the SLS process when printing with a polymer-fiber blend. Though advantages can be found by optimizing material properties by adding discontinuous fibers, adding fibers can also deteriorate material properties and increase the anisotropy of the final part. This study characterizes the effects of sintering PA12-CF blends to include fiber orientation and fracture analysis, and quantifies tensile data for 0%, 2.5%, 5%, and 10% volume CF in both horizontal and vertical print orientations. The novel results indicate the feasibility of tuning E based on %vol CF, leading to future work in SLS AM with CF fractions above 10%, and achieving precisely tuned functionally graded materials through SLS.

## 2. Materials and Methods

Material blends were tested for powder-fiber flowability, spreadability, and sinterability. PA12 tensile specimens were printed with blends of 0% CF (neat PA12), 2.5% CF, 5% CF, and 10% CF by volume (%vol). Density specimens were printed with blends of 0% CF, 5% CF, and 10% CF by volume. A minimum of three specimens from each blend were used for tensile and density characterization.

### 2.1. Material Preparation

Neat PA12 powder was sourced from Sintratec (Sintratec, Switzerland), and CF was sourced from Carbiso™ (Coseley, UK). Powder re-use has been found to affect particle coalescence and final part properties [37,38]. To minimize variability between prints, only fresh (unused) powder was used. Exact powder chemistry and geometry are proprietary, but tested particles were generally <0.1 mm in any direction. Fiber was rod-shaped, recycled, milled M80 CF with a 0.08 mm maximum length and 0.007 mm diameter. All material was sieved using a #100 SS mesh sieve (ASTME 1, Hogentogler Inc., Columbia, MD, USA) before blending.

Blend ratios were based on material density and were weighed for mixing ratios. Percent volume was calculated and used for reporting. All percentages of CF given in this report refer to percent volume. Materials were blended using a Reveo vacuum-sealed mechanical tumbler (FeraDyne, Superior, WI, USA). Blends loaded into the reservoir were smoothed and de-aerated but not packed.

### 2.2. Additive Manufacturing

A novel SLS printer with fully customizable parameters [39] was used to manufacture all test specimens. The SLS printer employed a 5W blue diode laser with a wavelength of 455 nm. The printing chamber maintained ambient air and pressure at elevated temperatures up to 162 °C.

The same recoating and hatch schemes were used for all specimens to increase consistency when comparing specimens. Recoating was accomplished with a single-blade metal recoater using a multi-pass recoating algorithm. The multi-pass recoating delivered double the required material layer height to the build area on the first right-to-left recoating pass. The recoater subsequently removed a single layer of powder on a second left-to-right pass. The multi-pass recoating aided in distributing a fully dense, homogenous 0.1 mm build layer on the build plate. A single-window, constant-time hatch pattern with perimeters and layer rasters oriented at 0 and 90 degrees was used. The multi-pass lasing scheme consisted of 3 laser passes over the part cross-section of each layer. The first laser pass preheated the current cross-section area. The second pass sintered the current layer and the third pass heat-treated (“baked”) the layer. Multi-pass recoating and lasing were particularly beneficial in decreasing porosity and warping while fully sintering higher fiber content blends.

**Figure 2:**
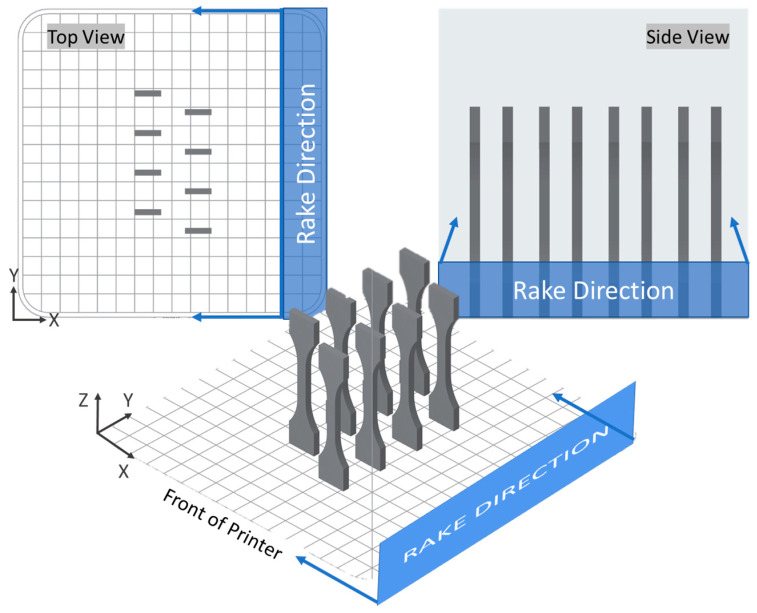
Vertical tensile specimen print scheme and powder recoating direction.

ASTM D638-14 Type V specimens modified similar to Lumpe et al. [40] and Tang et al. [41] were printed. Tensile specimens for each blend were printed in sets of 3–6 for horizontal specimens and sets of 6–12 vertical specimens with horizontal specimens offset in the y-direction and vertical specimens offset in x- and y-directions on the build plate (Figure 2) to improve powder flow and spreading uniformity. Separate density specimens were printed between the horizontal tensile specimens. The top layer powder temperature was maintained between 150 and 162 °C depending on print time. Laser power was maintained at 5 W with speeds for each blend given in Table 1.

### 2.3. Material Characterization

A qualitative evaluation of blends up to 40%vol CF blends was conducted to evaluate spreading and printing feasibility. Particle coalescence, layer bonding, and porosity were quantitatively evaluated using hardness, density, and tensile tests. Loose bulk density was evaluated per ASTM D7481 [42]. Hardness was evaluated using a shore D hardness test with three tests conducted on each specimen. Density tests were conducted on separate density specimens, and hardness tests were conducted on the grip portion of the tensile specimens for 0%, 5%, and 10% volume CF blends.

Tensile characterization included an evaluation of modulus of elasticity (E), yield stress (σ_y_), and ultimate tensile strength (UTS) for 0%, 2.5%, 5%, and 10% volume CF blends. XT wedge grips and an eXpert 5603 universal testing machine (ADMET Inc., Norwood, MA, USA) controlled by MTEST Quattro Material Testing System v5.00.02 (ADMET Inc., Norwood, MA, USA) were used for tensile testing. Grip rate was 10 mm/min, and ambient test temperature was ~23 °C in accordance with ASTM D638-14. Data collection rate was 100 Hz. An SM-1000 load cell (Interface Inc., Scottsdale, AZ, USA) was used to measure force. Position was measured by the eXpert 5603’s motor encoder. Strain was measured by a 3442 axial extensometer (Epsilon Technology Corp., Jackson, WY, USA).

Microscopy was accomplished using a Hitachi TM1000 variable pressure scanning electron microscope (SEM) (Hitachi High-Tech, Ibaraki, Japan) and a Dino-Lite Edge Plus (AM4917MZT) 1.3 MP 20x–220x (Dunwell Tech Inc., Torrance, CA, USA) microscope. A razor blade was used to section specimens, and SEM microscopy of cross-sections was used to analyze porosity and fiber distribution of each tested blend. Images of powder, fiber, and fracture surfaces were used to evaluate powder coalescence, voids, and fiber orientation qualitatively. Depth of field capability of ~0.5 mm at 220x magnification was used for porosity and fracture imaging [43].

## 3. Results

**Figure 3:**
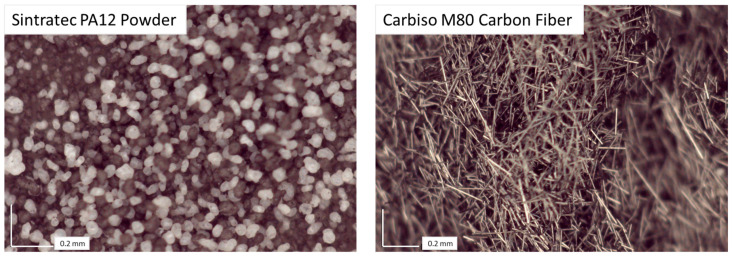
Neat Sintratec powder (**left**) and Carbiso M80 recycled powder (**right**).

**Figure 4:**
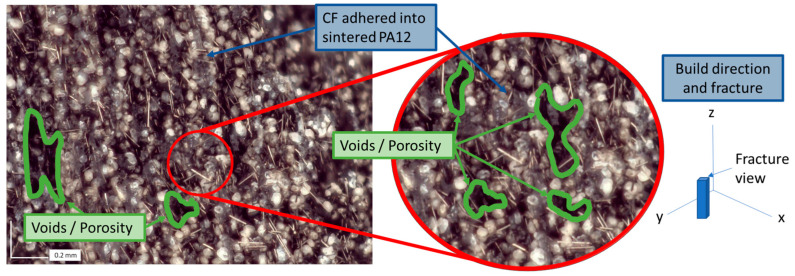
PA12 + 40% volume CF fracture surface showing CF sintered into PA12 particles with a high porosity volume fraction due to inconsistent spreading and blend clumping.

Images of unsintered neat powder show a blend of different shades of grey oval particles (Figure 3). Images of Carbiso M80 CF show relatively constant length rod-shaped fibers (Figure 3). Printing with these materials with 40%vol CF is possible but spreading inconsistencies result in high porosity (Figure 4). Blends of 20–40%vol are much more consistent but still have considerable porosity even with multi-pass recoating. Thus, quantitative testing was conducted on blends up to 10%vol CF.

**Figure 5:**
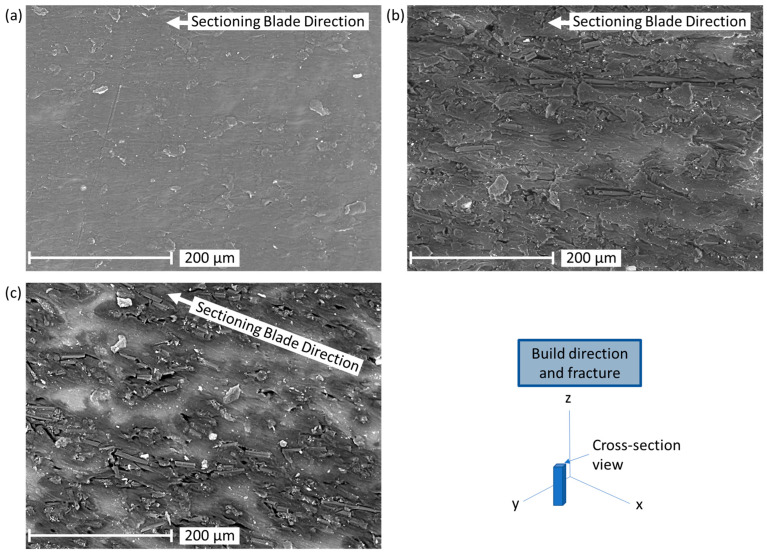
SEM images of cross-sections: (**a**) PA12 + 0%vol CF, (**b**) PA12 + 5%vol CF, (**c**) PA12 + 10%vol CF.

SEM images of specimen cross-sections provide insight into the fiber dispersion and porosity for 0, 5, and 10%vol CF blends (Figure 5). The cross-sections indicate minimal porosity with some damage to the matrix material that appears to be caused by the sectioning blade. The blade also appears to have oriented and damaged some of the fibers but gives insight into fiber density and dispersion. There appears to be some clumping in the fibers with areas of varying fiber density in both the 5 and 10%vol CF blends.

Density and hardness results were quantitatively analyzed for 0, 5, and 10%vol CF blends and are shown in Table 2 and Table 3, respectively. Sintratec reports 0.98 g/cm^3^ printed part density and 76 shore D hardness [44]. Carbiso reports a fiber density of 1.8 g/cm^3^ [45]. The multi-pass recoating and lasing used in this study, techniques that are not used natively in Sintratec SLS printers, likely increased printed density as compared to what was reported by Sintratec (~4% less). The “calculated” density in Table 2 assumes ideal mixing and sintering with no effects from material processing and no void formation. The theoretical increases in density for 5 and 10%vol CF blends were calculated using the measured neat PA12 density (1.024 g/cm^3^) and the CF density (1.8 g/cm^3^) reported by the manufacturer. Low porosity and consistency between prints are indicated by a low standard deviation in density (<0.005 g/cm^3^) and the close correlation between measured and calculated density (Table 2). Hardness also increased with an increase in CF (Table 3), and the measured neat hardness is within 2% of Sintratec’s reported Shore D hardness of 76 [44].

**Figure 6:**
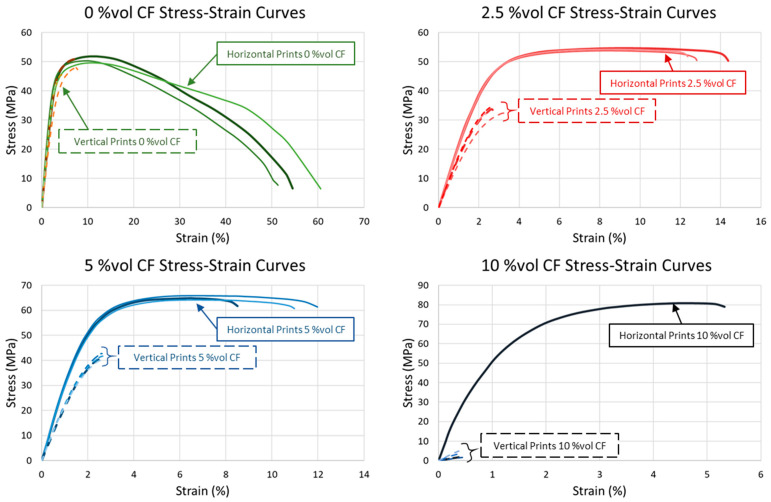
Stress–strain curves for 0, 2.5, 5, and 10% volume CF horizontal (solid lines) and vertical (dashed lines) specimens. (Note: Axis scaling is adjusted to highlight the difference between horizontal and vertical specimens of each CF blend. See Figure 7 with a common axis for all blends to compare tensile data between blends).

**Figure 7:**
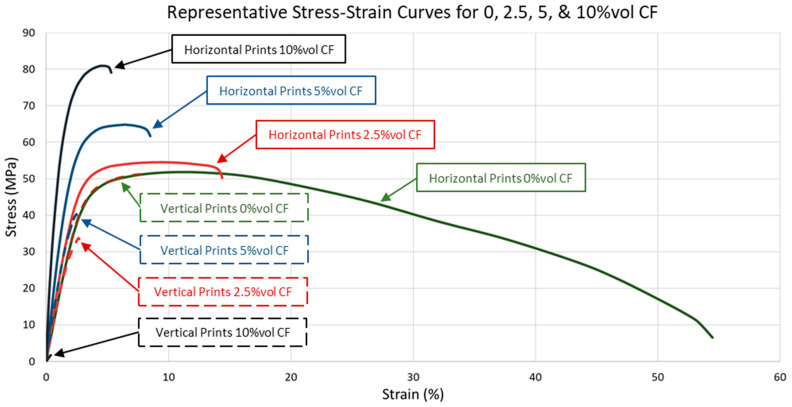
Representative Stress–strain curves for 0, 2.5, 5, and 10% volume CF horizontal (solid lines) and vertical (dashed lines) specimens.

**Figure 8:**
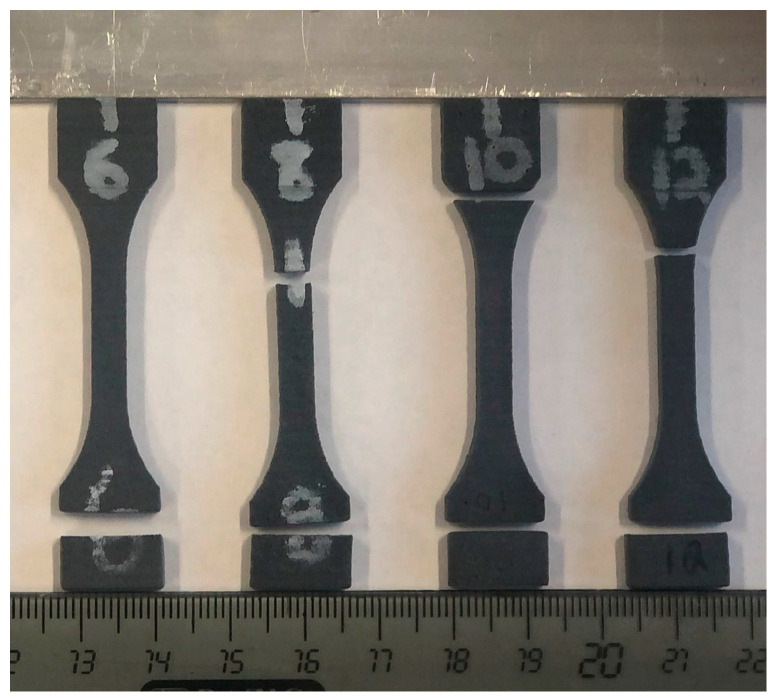
Example of post-print cleaning of 10%vol CF vertically printed tensile specimens.

Tensile properties are provided in Table 4, and stress–strain curves are shown in Figure 6 and Figure 7. Each graph in Figure 6 was scaled for each blend to more clearly depict the differences in load response for vertical and horizontal orientations for each composition. To provide a consistent scale and a more direct comparison between blends, representative stress–strain curves for each orientation and composition are presented in Figure 7. Tensile properties differed substantially between horizontal and vertical prints as CF content increased. Horizontal specimen modulus of elasticity, yield stress, and ultimate tensile strength consistently increased, as expected, with increased CF. Horizontal specimens with 2.5%vol CF had a minimal increase between blend ratios compared to the differences between 0, 5, and 10%vol CF. Tensile testing revealed that vertical specimens with CF have severe layer adhesion inconsistencies. This was partially due to longer print times for the vertical specimens. Print times for the horizontal specimens were 1–1.5 h and were 15–25 h (depending on laser speed and number of samples in a single print) for vertical specimens. The long vertical specimen print times periodically resulted in reservoir powder degradation. Degradation was most severe with 10%vol CF blends. Most vertical 10%vol CF specimens broke along a layer during post-print cleaning (Figure 8). Many others broke during grip tightening at the start of tensile testing or withstood minimal extension before fracturing along a layer. However, interaction between the fibers also appeared to play a key role in specimen tensile properties as fiber content was increased, resulting in a high failure rate of 10%vol CF specimens. Tensile test data was calculated for specimens with at least 0.30% strain. All but one horizontal 10%vol CF specimen failed during post-print processing or before 0.30% strain during tensile tests.

**Figure 9:**
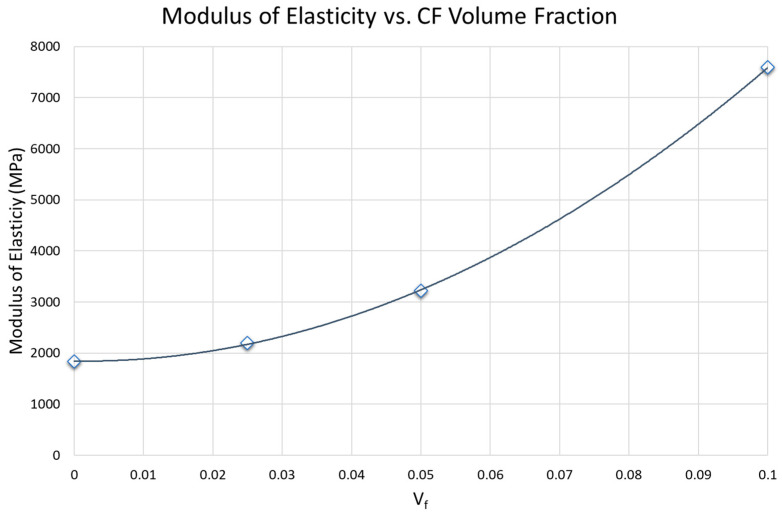
Plot of Modulus of Elasticity for the horizontal orientation vs. %vol CF data points with Equation (1) [$E=590072{\left(V_{f}\right)}^{2}-1586.9(V_{f})+1843.3$] curve.

Horizontally printed specimen modulus of elasticity was specifically analyzed based on CF Volume Fraction (V_f_). E vs. %CF was graphed (Figure 9) with a 2nd order polynomial curve fit (Equation (1)).

(1) $$E=590072{\left(V_{f}\right)}^{2}-1586.9(V_{f})+1843.3$$

The constants in Equation (1) result in units of MPa for E as seen in Figure 9. The root mean square error (RMSE) of the data points to the curve is 19.8. V_f_ is bounded by the measured data, and results should not be extrapolated beyond a volume fraction of 0.1 (10%vol CF).

**Figure 10:**
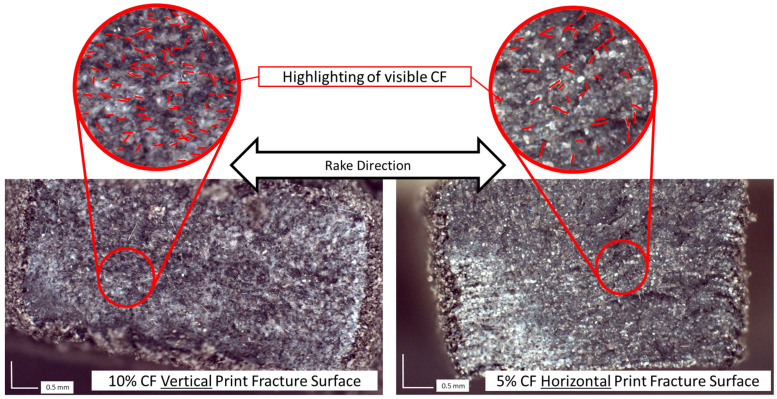
Fracture surface of 10% volume CF (**left**) and 5% volume CF (**right**) tensile specimens. Red lines indicate visible fibers on surface.

Fractography indicates a relatively even distribution of fibers (Figure 10). However, this was only a visual and qualitative inspection. The CF and sintered PA12 are similar in color in these images, and it was not possible to autonomously distinguish the fibers from the sintered PA12 using ImageJ contrast imaging software (version 1.54i, U. S. National Institutes of Health, Bethesda, MD, USA, https://imagej.net/ij/) quantify CF content. The fibers observed on fracture surfaces are more aligned along the recoating direction for the vertical prints than the horizontal prints. The images also showed more fiber pullout for the vertical specimens than the horizontal specimens.

## 4. Discussion

Solid-state particle blending has unique challenges and potential advantages compared to liquid or molten-state blending. Mechanically blending powders and fillers allows material ratios to be modified at any time through the blending process and shifts flowability requirements from the liquid to the powder state. This shift allows relatively simple powder/fiber blending at any ratio to tune final material properties without recreating new stock material (such as filament-based MEX) each time a new matrix/fiber ratio is needed. However, maintaining a homogeneous mixture throughout the PBF process can be difficult if the particle mechanical and geometric properties differ within the blend.

The way in which PBF deposits polymer and fiber in a solid state may enable printing with fiber content much greater than can be achieved with processes that rely upon material deposition through fluid flow or in the molten state, such as filament-based thermal reaction bonding MEX (MEX-TRB/F, also commonly known as Fused Filament Fabrication or Fused Deposition Modeling). With some exceptions [46,47], studies investigating MEX-TRB/F with composites are often limited to 20%vol discontinuous CF or less due to the high viscosity of fiber-rich blends. Some MEX-TRB/F method studies have increased fiber volume by using continuous fiber [48]. This study showed powder spreading and sintering were possible up to 40%vol CF but blends were limited to 10%vol CF because of the high porosity with higher fiber fractions. Achieving final parts with 40%vol fiber content would be advantageous for many applications, but using the tested methods, high void content above 10%vol CF greatly limits the mechanical properties of such composites. Hence, the mechanical powder spreading during PBF may limit the effective range of mixtures even if printer operations sinter a structural part at these fiber volume fractions. Further testing is recommended with different recoating methods (possibly using multi-pass recoating with a roller) and different shape powders and fibers (to include encapsulated fibers) to investigate achieving fully dense parts with fiber ratios > 10%vol.

The density and hardness measured from mixtures up to 10%vol CF content correlate well with published data from the material manufacturers [44,45], suggesting consistency between prints with low porosity as indicated by a low standard deviation in porosity (<0.005 g/cm^3^) and close correlation to the calculated density (Table 2). However, calculated density (Table 2) should not be considered absolute density (100% dense with zero porosity). Microscopy of these parts with CF up to 10%vol correlated well with the density results and indicated very low porosity with fully coalesced particles (Figure 5 and Figure 10). Conversely, microscopy of 40%vol specimens indicated good fiber-particle adhesion but with significant voids. The measured density generally increased proportionally with an increase in CF as expected, but some small differences above and below the calculated density indicate that there may be variability in voids, crystallinity, shrinkage, and mixing. The finely tuned processes in this study can be expected to increase crystallinity and therefore increase shrinking which may explain the higher density observed in the 5%vol CF. However, further testing is recommended using different lasers (primarily varying wavelength and power), hatch patterns, and recoating methods to increase particle coalescence and decrease porosity at high CF ratios. This is particularly critical in vertical print sections where load-bearing capability relies on particle coalescence bonding the layers.

Anisotropy has also long plagued AM and SLS [10,11,12,13], and the current results show that adding fibers exacerbates this problem. Tensile properties increased in both modulus of elasticity and UTS with increased CF content. Vertically oriented specimens tended to have degraded material properties with increased CF content. These results seem to indicate that as CF content increases, there are more locations of CF touching CF, which will not bond during the sintering process. The CF-CF interface effectively acts as a void and stress concentrator. This is most evident in the brittleness and fracturing of the 10%vol CF vertical samples in Figure 7 and Figure 8. However, similar trends were seen in the 10%vol CF horizontal specimens with only a single specimen achieving over a 0.3% strain. This emphasizes the importance of future research to consistently manufacture CFRPs with CF regardless of print orientation. Slower laser speeds and higher print times were necessary for 10%vol CF blends since they require the highest energy density for sintering. To decrease overall print time, it is recommended to incorporate multi-pass laser testing with a higher-powered laser. Encapsulating the CF with PA12 matrix material could also decrease the CF-CF effect, but at the same time greatly complicates stock material manufacturing and mixing specific ratios. Current research on CRFPs manufactured via more traditional resin layup methods has shown that CF surface treatments and matrix nanofillers can improve fiber-matrix bonding and decrease fiber-fiber slippage [49,50]. However, surface treatments can be hazardous to the environment and require complex equipment and conditions to accomplish. In contrast, nanofillers can offer a more economical method to increase overall composite properties by improving the interfacial properties between the fiber and polymer interfaces of CFRP. Future work is still required to expand these methods to SLS manufacturing making it prudent to continue investigating incorporating both coated and non-coated CF in SLS manufacturing.

Fractography was helpful in evaluating fiber orientation, but distinguishing between fiber, sintered particles, and voids was difficult optically since all three features are dark in color. Analysis through micro-computed tomography with image segmentation [51] is recommended to better characterize the composite structures, including fiber orientation and void content. The deformation from sectioning the samples and from failure affected the fiber orientation observed in microscopy (Figure 5 and Figure 10). This was especially true for horizontal specimen fracture surfaces. The fiber orientation appeared to be less affected by fracture in vertical specimens. This likely indicates that fibers share a higher proportion of load in structures experiencing horizontal loading, as indicated by the trends observed in modulus of elasticity and UTS.

Horizontally printed specimens demonstrate the possibility of customizing material properties with the addition of CF (Figure 9). The rule of mixtures is often an effective method to estimate expected material properties in composites. However, since the fibers are not perfectly aligned with the load (longitudinal direction) or perpendicular to the load (transverse direction) key assumptions for either iso-strain or iso-stress are not met. A reinforcement efficiency factor could be used to compensate for neither iso-strain nor iso-stress conditions being met [52] (often 3/8 for fibers randomly and uniformly distributed within a specific plane and 1/5 for fibers randomly and uniformly distributed within three-dimensional space [53]). A modified rule of mixtures equation (MROM) is often used for discontinuous short fiber CFRPs given by the following equation:

(2) $$E=\eta_{0}\eta_{L}E_{f}V_{f}+E_{m}V_{m}$$

where E is the modulus of the composite, E_f_ and E_m_ are the moduli of the fiber and matrix, respectively, and V_f_ and V_m_ are the volume fractions of the fiber and matrix, respectively. The Krenchel orientation factor is η_0_, and η_L_ is the Cox shear lag factor [54]. The MROM is a linear relation between the measured matrix and fiber modulus but a 4th-order relationship to the orientation tensor found in η_0_ which is very difficult to measure [55]. Some success has been found simply estimating η_0_ = 0.2 for random fibers in all three dimensions and resulting crack propagation around the CF (as opposed to through the CF) [56,57]. However, key assumptions are not met with SLS AM since the fibers are clearly semi-oriented along the spreading direction (Figure 10). The degree of orientation also appears to change with fiber density with increasing orientation as density increases. Due to the difficulty of measuring and calculating the η_0_, there have been attempts to determine η_0_ indirectly in AM parts by printing CFRP tensile specimens and first measuring the total composite stiffness [58]. The findings of this research indicate a more simplistic approach using experimental data from printed specimens but again assume a linear relationship. The data from the present work suggests that a non-linear approach using a second-order polynomial fit solely based on V_f_ (Equation (1)) may better predict certain printed part moduli for specific materials and print settings. The printing process is complex and the non-linearity we found is likely caused by multiple interacting factors, but may primarily be due to changes in fiber-fiber interaction and fiber orientation based on V_f_. This indicates that this relationship is dependent not only on material blending but also on recoating and lasing techniques. This may lead to each experimentally determined constant in Equation (1) being specific to SLS AM equipment and process settings. Also, though the experimentally determined 2nd order relationship (Equation (1)) has a low RMSE value indicating the feasibility of using a non-linear approach based on V_f_ to tune E; it is extremely important to note that many more data points at varying CF blends are required to verify these results. Additionally, these results should not be extrapolated beyond the maximum tested CF density (10%vol) until testing is conducted above this value. Future testing is recommended to not only verify the E vs. CF trend and compare this to established trends like the MROM, but also to quantify other material property trends as a function of V_f_.

## 5. Conclusions

Discontinuous CF can be a powerful tool when added to a polymer matrix. However, adding CF to PBF can increase the already anisotropic process, which must be considered when integrating CF into AM. PA12/CF blends up to 10%vol CF appear to have “tunable” material properties for horizontal blends, offering a promising future for mechanical blending in PBF. In contrast, vertically printed specimens above 5%vol had very poor tensile properties. Further research is recommended with different sintering energy sources and different blends to more thoroughly characterize the tunability of material properties using mechanically blended powders and fibers. Continued research should be performed to develop techniques to increase printability while decreasing anisotropy in parts with high CF content. This should include a two-pronged approach looking at both the stock materials and SLS methods to build on current SLS composite research [59]. Material research should include fiber coatings and matrix fillers compatible with the SLS process. SLS method research should include powder application techniques involving different spreading devices (i.e., counter-rotating rollers) and multiple recoaters (i.e., one roller in the x-direction and a second roller in the y-direction) to align fibers in pre-planned directions.

The datasets and results from this research provide a mechanism to tune material properties and increase the functionality of fiber-reinforced prints through print orientation selection. Initial results indicate a relatively simplistic and useful trend between CF content and modulus of elasticity (Equation (1)); however, testing more blend ratios is required to establish this relationship fully. Though stiffness and strength were the primary properties investigated, functionalizing polymers with CF can have other benefits, such as wear resistance and thermal resistance. Further testing is recommended to analyze possible trends between other material properties and CF ratios. Relationships between material properties and CF ratios are particularly powerful when customizing material properties and manufacturing functionally graded materials. The feasibility of manufacturing functionally graded materials via PBF has been established [59,60,61,62]. Tunable materials with specific relationships between fiber content and material properties could be combined with multi-material PBF systems to manufacture highly customizable functionally graded parts.

## Figures and Tables

**Table 1:** Preheat, sinter, and bake (post-heat) laser speeds for each blend.

Blend	Print Orientation	Laser Speeds
(%vol CF)		Preheat-Sinter-Bake (mm/s)
0	Horizontal Vertical	2000-500-3000 1500-800-2000
2.5	Horizontal Vertical	*-500-2000 *-400-1200
5	Horizontal Vertical	1000-400-1500 1000-400-1500
10	Horizontal Vertical	2000-400-3000 1000-400-1500

* Preheat lasing not used for 2.5%vol CF specimens.

**Table 2:** PA12/CF buoyancy density results.

Blend	Measured Density	Calculated Density	
(%vol CF)	(g/cm^3^) ± SD	(g/cm^3^)	% Difference
0	1.024 ± 0.0009	1.024	NA
5	1.076 ± 0.0047	1.063	1%
10	1.100 ± 0.0007	1.102	0%

**Table 3:** PA12/CF Shore D hardness results.

Blend	Measured Hardness
(%vol CF)	(Shore D Rating) ± SD
0	74.67 ± 1.48
5	77.89 ± 0.86
10	81.17 ± 0.66

**Table 4:** PA12/CF tensile properties.

Blend		Modulus	Yield Stress	Yield Strain	UTS	Failure Strain
(%vol CF)	Orientation	(MPa) ± SD	(MPa) ± SD	(%) ± SD	(MPa) ± SD	(%) ± SD
0	Horizontal	1832 ± 171	36.41 ± 2.04	2.20 ± 0.08	51.24 ± 0.75	52.85 ± 2.23
	Vertical	1666 ± 286	33.17 ± 0.69	2.24 ± 0.39	46.07 ± 1.65	4.94 ± 2.46
2.5	Horizontal	2203 ± 65	37.04 ± 1.12	1.88 ± 0.10	54.29 ± 0.49	13.18 ± 1.06
	Vertical	1809 ± 165	24.69 ± 1.24	1.57 ± 0.10	33.13 ± 1.03	2.68 ± 0.38
5	Horizontal	3217 ± 104	41.26 ± 1.50	1.48 ± 0.07	65.05 ± 0.80	10.49 ± 1.79
	Vertical	2412 ± 81	29.46 ± 1.94	1.42 ± 0.11	41.33 ± 1.36	2.58 ± 0.08
10	Horizontal	7589 ± *	42.09 ± *	0.75 ± *	80.46 ± *	5.23 ± *
	Vertical	854 ± 490	3.35 ± 2.33	0.44 ± 0.10	3.35 ± 2.33	0.44 ± 0.10

* Only a single horizontal 10 %vol CF specimen did not fail during post-processing and strained >30%.

## Data Availability

Data is contained within the article.

## Abbreviations

The following abbreviations are used in this manuscript:

AM	Additive Manufacturing
CF	Carbon Fiber
CFRP	Carbon Fiber-Reinforced Polymer Composites
HA	Hydroxyapatite
E_f_	Fiber Modulus of Elasticity
E_m_	Matrix Modulus of Elasticity
FGM	Functionally Graded Material
MPa	Megapascal
MEX	Material Extrusion
MROM	Krenchel and Cox Modified Rule of Mixtures Equation
Nm	Nanometer
PA11	Polyamide 11
PA12	Polyamide 12
PEEK	Polyetheretherketone
PBF	Powder Bed Fusion
RSME	Root Mean Square Error
SD	Standard Deviation
SLS	Selective Laser Sintering
TRB/F	Thermal Reaction Bonding with Spooled Filament-Based Stock Material
T_g_	Glass Transition Temperature
T_m_	Melting Temperature
T_r_	Re-crystallization Temperature
V_f_	Fiber Volume Fraction
V_m_	Matrix Volume Fraction
%vol	Percent Volume

## References

[1] Bhatia A., Sehgal A.K. (2021). Additive manufacturing materials, methods and applications: A review. Mater. Today Proc..

[2] Vanaei S., Elahinia M. (2024). Applicable Materials and Techniques in 3D Printing, Industrial Strategies and Solutions for 3D Printing: Applications and Optimization.

[3] Vanaei H.R., Khelladi S., Tcharkhtchi A. (2023). Polymers and Their Application in 3D Printing.

[4] Schmid M., Wegener K. (2016). Additive Manufacturing: Polymers Applicable for Laser Sintering (LS). Procedia Eng..

[5] Razaviye M.K., Tafti R.A., Khajehmohammadi M. (2022). An investigation on mechanical properties of PA12 parts produced by a SLS 3D printer: An experimental approach. CIRP J. Manuf. Sci. Technol..

[6] Gomes P.C., Piñeiro O.G., Alves A.C., Carneiro O.S. (2022). On the Reuse of SLS Polyamide 12 Powder. Materials.

[7] Tomanik M., Żmudzińska M., Wojtków M. (2021). Mechanical and Structural Evaluation of the PA12 Desktop Selective Laser Sintering Printed Parts Regarding Printing Strategy. 3D Print. Addit. Manuf..

[8] Gavcar B., Sumer E.H., Sagbas B., Katiyar J.K. (2023). Effect of build orientation on the green tribological properties of multi-jet fusion manufactured PA12 parts. Proc. Inst. Mech. Eng. Part J J. Eng. Tribol..

[9] Cai C., Tey W.S., Chen J., Zhu W., Liu X., Liu T., Zhao L., Zhou K. (2020). Comparative study on 3D printing of polyamide 12 by selective laser sintering and multi jet fusion. J. Mech. Work. Technol..

[10] Zárybnická L., Petrů J., Krpec P., Pagáč M. (2022). Effect of Additives and Print Orientation on the Properties of Laser Sintering-Printed Polyamide 12 Components. Polymers.

[11] Calignano F., Giuffrida F., Galati M. (2021). Effect of the build orientation on the mechanical performance of polymeric parts produced by multi jet fusion and selective laser sintering. J. Manuf. Process..

[12] Zohdi N., Yang R. (2021). Material anisotropy in additively manufactured polymers and polymer composites: A review. Polymers.

[13] El Magri A., Bencaid S.E., Vanaei H.R., Vaudreuil S. (2022). Effects of Laser Power and Hatch Orientation on Final Properties of PA12 Parts Produced by Selective Laser Sintering. Polymers.

[14] Arai S., Tsunoda S., Yamaguchi A., Ougizawa T. (2019). Effect of anisotropy in the build direction and laser-scanning conditions on characterization of short-glass-fiber-reinforced PBT for laser sintering. Opt. Laser Technol..

[15] Chen H., Zhu W., Tang H., Yan W. (2021). Oriented structure of short fiber reinforced polymer composites processed by selective laser sintering: The role of powder-spreading process. Int. J. Mach. Tools Manuf..

[16] Chung H., Das S. (2006). Processing and properties of glass bead particulate-filled functionally graded Nylon-11 composites produced by selective laser sintering. Mater. Sci. Eng. A.

[17] Chung H., Das S. (2008). Functionally graded Nylon-11/silica nanocomposites produced by selective laser sintering. Mater. Sci. Eng. A.

[18] Jepson L.R., Beaman J.J., Bourell D.L., Wood K.L. Multi-Material Selective Laser Sintering: Empirical Studies and Hardware Development. Proceedings of the NSF Design and Manufacturing Grantees Conference.

[19] Wei C., Sun Z., Chen Q., Liu Z., Li L. (2019). Additive Manufacturing of Horizontal and 3D Functionally Graded 316L/Cu10Sn Components via Multiple Material Selective Laser Melting. J. Manuf. Sci. Eng..

[20] Tan K., Chua C., Leong K., Cheah C., Cheang P., Abu Bakar M., Cha S. (2003). Scaffold development using selective laser sintering of polyetheretherketone–hydroxyapatite biocomposite blends. Biomaterials.

[21] Green J.T., Rybak I.A., Slager J.J., Lopez M., Chanoi Z., Stewart C.M., Gonzalez R.V. (2023). Local composition control using an active-mixing hotend in fused filament fabrication. Addit. Manuf. Lett..

[22] Quero R.F., Costa B.M.d.C., da Silva J.A.F., de Jesus D.P. (2022). Using multi-material fused deposition modeling (FDM) for one-step 3D printing of microfluidic capillary electrophoresis with integrated electrodes for capacitively coupled contactless conductivity detection. Sens. Actuators B Chem..

[23] Baca D., Ahmad R. (2020). The impact on the mechanical properties of multi-material polymers fabricated with a single mixing nozzle and multi-nozzle systems via fused deposition modeling. Int. J. Adv. Manuf. Technol..

[24] Wang Z., Wang L., Tang F., Chen J. (2024). Multi-material additive manufacturing via fused deposition modeling 3D printing: A systematic review on the material feeding mechanism. Proc. Inst. Mech. Eng. Part E J. Process. Mech. Eng..

[25] Patpatiya P., Chaudhary K., Shastri A., Sharma S. (2022). A review on polyjet 3D printing of polymers and multi-material structures. Proc. Inst. Mech. Eng. Part C J. Mech. Eng. Sci..

[26] Chadha C., Olaivar G., Patterson A.E., Jasiuk I.M. Design for Multi-Material Manufacturing Using Polyjet Printing Process: A Review. Proceedings of the ASME Design Engineering Technical Conference.

[27] Tee Y.L., Peng C., Pille P., Leary M., Tran P. (2020). PolyJet 3D Printing of Composite Materials: Experimental and Modelling Approach. JOM.

[28] Yan C., Hao L., Xu L., Shi Y. (2011). Preparation, characterisation and processing of carbon fibre/polyamide-12 composites for selective laser sintering. Compos. Sci. Technol..

[29] Yuan S., Shen F., Chua C.K., Zhou K. (2019). Polymeric composites for powder-based additive manufacturing: Materials and applications. Prog. Polym. Sci..

[30] Tobolsky A., Callinan T. (1960). Properties and structure of polymers. J. Electrochem. Soc..

[31] AZOM (2001). Thermoplastics-An Introduction, AZO Materials. https://www.azom.com/article.aspx?ArticleID=83.

[32] Weber R., Hafner M., Michalowksi A., Graf T., Emmelmann C. (2012). Influence of laser cutting parameters on CFRP part quality. J. Mach. Tools Manuf..

[33] Rodríguez A.G., Mora E.E., Velasco M.A., Tovar C.A.N. (2023). Mechanical properties of polyamide 12 manufactured by means of SLS: Influence of wall thickness and build direction. Mater. Res. Express.

[34] Cano A., Salazar A., Rodríguez J. (2018). Effect of temperature on the fracture behavior of polyamide 12 and glass-filled polyamide 12 processed by selective laser sintering. Eng. Fract. Mech..

[35] Slager J.J., Earp B.C., Ibrahim A.M. (2024). Influence of Build Orientation and Part Thickness on Tensile Properties of Polyamide 12 Parts Manufactured by Selective Laser Sintering. Polymers.

[36] Slager J.J. (2022). Multi-Material/Fiber/Particulate Selective Laser Sintering System Capable of Local Composition Control and Material Gradients. Ph.D. Thesis.

[37] Dadbakhsh S., Verbelen L., Verkinderen O., Strobbe D., Van Puyvelde P., Kruth J.-P. (2017). Effect of PA12 powder reuse on coalescence behaviour and microstructure of SLS parts Special Issue “Trends and Prospects of Biomedical Alloys in Additive Manufacturing” in applied sciences with impact factor of 2.474 View project Dimensional Me-trology CMM View project Effect of PA12 powder reuse on coalescence behaviour and microstructure of SLS parts. Eur. Polym. J..

[38] Pavan M., Faes M., Strobbe D., Van Hooreweder B., Craeghs T., Moens D., Dewulf W. (2017). On the influence of inter-layer time and energy density on selected critical-to-quality properties of PA12 parts produced via laser sintering. Polym. Test..

[39] Slager J., Green J. (2024). Multi-Material Powder Bed Fusion. U.S. Patent.

[40] Lumpe T.S., Mueller J., Shea K. (2019). Tensile properties of multi-material interfaces in 3D printed parts. Mater. Des..

[41] Tang H., Chen H., Sun Q., Chen Z., Yan W. (2021). Experimental and computational analysis of structure-property relationship in carbon fiber reinforced polymer composites fabricated by selective laser sintering. Compos. Part B Eng..

[42] Chatham C.A., Long T.E., Williams C.B. (2019). A review of the process physics and material screening methods for polymer powder bed fusion additive manufacturing. Prog. Polym. Sci..

[43] Dino-Lite Digital Microscope. https://www.dino-lite.com/faq_detail.php?index_id=34.

[44] (2022). SINTRATEC PA12, Sintratec Material Data Sheet. https://www.solidpro.de/wp-content/uploads/3D-Druck/Sintratec/Datenblatt/Sintratec-Datenblatt-PA12-EN.pdf.

[45] CARBISOTM MF Milled Fiber—ELG CARBON FIBRE LTD.-PDF Catalogs|Technical Documentation|Brochure. https://pdf.aeroexpo.online/pdf/elg-carbon-fibre-ltd/carbiso-mf-milled-fiber/182993-7284.html.

[46] Tekinalp H.L., Kunc V., Velez-Garcia G.M., Duty C.E., Love L.J., Naskar A.K., Blue C.A., Ozcan S. (2014). Highly oriented carbon fiber–polymer composites via additive manufacturing. Compos. Sci. Technol..

[47] Lu S., Zhang B., Niu J., Yang C., Sun C., Wang L., Li D. (2024). Effect of fiber content on mechanical properties of carbon fiber-reinforced polyether-ether-ketone composites prepared using screw extrusion-based online mixing 3D printing. Addit. Manuf..

[48] Parker M., Inthavong A., Law E., Waddell S., Ezeokeke N., Matsuzaki R., Arola D. (2022). 3D printing of continuous carbon fiber reinforced polyphenylene sulfide: Exploring printability and importance of fiber volume fraction. Addit. Manuf..

[49] Feng J., Gao C., Safaei B., Qin Z., Wu H., Chu F., Scarpa F. (2024). Exceptional damping of CFRPs: Unveiling the impact of carbon fiber surface treatments. Compos. Part B Eng..

[50] Joo J.H., Kim S.H., Yim Y.J., Bae J.S., Seo M.K. (2025). Interfacial Interlocking of Carbon Fiber-Reinforced Polymer Composites: A Short Review. Polymers.

[51] Sommacal S., Matschinski A., Holmes J., Drechsler K., Compston P. (2023). Detailed void characterisation by X-ray computed to-mography of material extrusion 3D printed carbon fibre/PEEK. Compos. Struct..

[52] Callister W.D., Rethwisch D.G. (2022). Fundamentals of Materials Science and Engineering: An Integrated Approach.

[53] Krenchel H. (1964). Fibre Reinforcement: Theoretical and Practical Investigations of the Elasticity and Strength of Fibre-Reinforced Materials.

[54] Cox H.L. (1952). The elasticity and strength of paper and other fibrous materials. Br. J. Appl. Phys..

[55] Hine P., Parveen B., Brands D., Caton-Rose F. (2014). Validation of the modified rule of mixtures using a combination of fibre orien-tation and fibre length measurements. Compos. Part A Appl. Sci. Manuf..

[56] Alshabib A., Jurado C.A., Tsujimoto A. (2022). Short fiber-reinforced resin-based composites (SFRCs); Current status and future perspectives. Dent. Mater. J..

[57] Norman D.A., Robertson R.E. (2003). The effect of fiber orientation on the toughening of short fiber-reinforced polymers. J. Appl. Polym. Sci..

[58] Adil S., Lazoglu I. (2023). A review on additive manufacturing of carbon fiber-reinforced polymers: Current methods, materials, mechanical properties, applications and challenges. J. Appl. Polym. Sci..

[59] Mussatto A. (2022). Research progress in multi-material laser-powder bed fusion additive manufacturing: A review of the state-of-the-art techniques for depositing multiple powders with spatial selectivity in a single layer. Results Eng..

[60] Mehrpouya M., Tuma D., Vaneker T., Afrasiabi M., Bambach M., Gibson I. (2022). Multimaterial powder bed fusion techniques. Rapid Prototyp. J..

[61] Bareth T., Binder M., Kindermann P., Stapff V., Rieser A., Seidel C. (2022). Implementation of a multi-material mechanism in a laser-based powder bed fusion (PBF-LB) machine. Procedia CIRP.

[62] Cortis D., Pilone D., Grazzi F., Broggiato G., Campana F., Orlandi D., Shinohara T., Planell O.S. (2024). Functionally graded material via L-PBF: Characterisation of multi-material junction between steels (AISI 316L/16MnCr5), copper (CuCrZr) and aluminium alloys (Al-Sc/AlSi10Mg). Prog. Addit. Manuf..

